# Evaluation of Free-Living Motor Symptoms in Patients With Parkinson Disease Through Smartwatches: Protocol for Defining Digital Biomarkers

**DOI:** 10.2196/72820

**Published:** 2025-07-28

**Authors:** Carlos Polvorinos-Fernández, Luis Sigcha, María Centeno-Cerrato, Guillermo de Arcas, Miriam Grande, Mayca Marín, Isabel Pareés, Juan Carlos Martínez-Castrillo, Ignacio Pavón

**Affiliations:** 1 Department of Mechanical Engineering, Instrumentation and Applied Acoustics Research Group ETSI Industriales Universidad Politécnica de Madrid Madrid Spain; 2 Department of Physical Education and Sports Science, Health Research Institute University of Limerick Limerick Ireland; 3 Data-Driven Computer Engineering (D2iCE) Research Centre University of Limerick Limerick Ireland; 4 Asociación Parkinson Madrid Madrid Spain; 5 Neurology Department, IRYCIS Hospital Ramón y Cajal Madrid Spain

**Keywords:** movement analysis, wearable devices, home monitoring, health indicator, smart health care

## Abstract

**Background:**

Monitoring motor symptoms in patients with Parkinson disease (PD) presents significant challenges due to the complex nature of symptom progression, variations in medication responses, and the fluctuations that can occur throughout the day. Traditional neurological visits provide only a limited perspective of a patient’s overall condition, with challenges in achieving accurate and objective assessments of symptoms. To bridge this gap, extended monitoring in nonclinical settings could play a critical role in personalizing treatments and improving their efficacy. Wearable devices have emerged as potential tools for assessing PD symptom severity; however, studies integrating both in-clinic and free-living conditions, as well as multiday monitoring, remain scarce. Defining digital biomarkers that provide valuable insights into motor symptoms could enable comprehensive monitoring and tracking of PD in various contexts, facilitating more precise medication adjustments and the implementation of advanced therapeutic strategies.

**Objective:**

This study aims to collect a dataset to support the proposal and definition of digital biomarkers of PD motor symptoms using wearable devices. Data will be collected both in a supervised setting and continuously in a remote, free-living context during participants’ normal daily activities; the study will include patients with PD and healthy controls. The goal is to identify reliable digital biomarkers that can effectively distinguish patients with PD from healthy controls and classify disease severity in both supervised and unsupervised free-living environments.

**Methods:**

This paper outlines a protocol for an observational case-control study aimed at assessing motor symptoms in patients with PD using a smartwatch. The smartwatch will record accelerometer, gyroscope, and physical activity data. Participants will be instructed to perform a series of exercises guided via a smartphone. Measurements will be collected in 2 settings: a supervised clinical environment, with motor symptoms assessments conducted at the beginning and end of the study, and in an unsupervised free-living context for 1 week. In both settings, participants will be required to wear the smartwatch while performing the same set of exercises. In their daily routine, participants will be required to wear the smartwatch continuously throughout the day, removing it only at night for charging.

**Results:**

Participant recruitment and data collection started in December 2024 and will continue until spring 2025. The study aims to enroll 20 participants with PD and 20 healthy controls.

**Conclusions:**

It is anticipated that the generation of a dataset of accelerometer and gyroscope signal data recorded from patients with PD at various stages of the disease, alongside data from a control group, will enable robust comparative and impactful analyses. In addition, the study seeks to develop analytical techniques capable of tracking PD symptoms in real-life scenarios, both in everyday settings and clinical environments.

**Trial Registration:**

ClinicalTrials.gov NCT06817772; https://clinicaltrials.gov/study/NCT06817772

**International Registered Report Identifier (IRRID):**

DERR1-10.2196/72820

## Introduction

### Background

In the context of an increasingly aging society characterized by an inverted population pyramid, the treatment and management of neurodegenerative diseases represent an important challenge. In 2013, the World Health Organization recognized neurodegenerative diseases as “a global health problem that must be addressed without delay for humanitarian, social, and economic reasons” [1]*.*

Parkinson disease (PD) exemplifies this issue, with the number of individuals affected rising by 250% between 1990 and 2015 [2]. In Spain, a new case of PD is diagnosed approximately every 45 minutes, with 15% of these cases occurring in individuals aged < 45 years [3]. PD is a progressive neurodegenerative disorder characterized by the selective degeneration of dopaminergic neurons in the substantia nigra pars compacta, which supports the corticosubcortical network, which facilitates limbic, associative, cognitive, and motor functions [4]. In early stages, PD often exhibits nonmotor dysfunctions, including impairments in working memory, executive function, attention, visuospatial capacity, and semantic fluency, which may eventually progress to dementia [5].

In advanced stages, motor symptoms (such as tremor, bradykinesia, postural instability, and rigidity) and nonmotor symptoms (including cognitive decline, mood disorders, sleep disturbances, and autonomic dysfunction) become more pronounced, significantly affecting patients’ quality of life [6,7].

Currently, there is no cure for PD. Treatment strategies typically involve pharmacological and surgical interventions, often complemented by nonpharmacological therapies [8]. Pharmacological treatments focus on restoring dopamine levels to preserve motor function for as long as possible [9]. While effective in managing hypokinesia (loss of movement) and tremor, these therapies remain beneficial within a therapeutic window of 5 to 10 years, after which 50% to 80% of patients develop dyskinesias (involuntary muscle movements). In addition, certain patients exhibit resistance to these treatments [10].

Nonpharmacological interventions are crucial for enhancing patients’ quality of life [11]. These include traditional treatments such as physical exercise, physiotherapy, occupational therapy, or cognitive training, among others [12-14]. Experimental approaches, including noninvasive neurostimulation techniques such as transcranial direct current stimulation [15], are also being explored for their potential to improve symptom management.

The diagnosis of PD is primarily clinical, relying on a detailed anamnesis based on information from the patient and their family, combined with a physical examination [16]. These consultations are used to conduct clinical assessments, adjust medications, and prescribe exercises or therapies. Several clinical scales support this process, with the Movement Disorder Society-Unified Parkinson’s Disease Rating Scale (MDS-UPDRS) [17] being the most widely used. However, the time allocated per patient during these visits is limited [18]. Reflecting this constraint, numerous resources, including guidelines, websites, and individualized workshops, have been developed to help patients and caregivers prepare effectively for their neurologist appointments [19-22].

Recent studies have highlighted the suboptimal accuracy of clinical diagnoses for PD, as the techniques are inadequate for capturing the nuanced and fluctuating nature of PD symptoms, which can vary significantly throughout the day [23]. Clinical assessments, conducted infrequently during brief consultations, fail to provide a comprehensive understanding of patients’ conditions in their everyday lives [24]. This limitation underscores the need for continuous monitoring tools to complement periodic clinical evaluations.

To enhance diagnostic resources for objective monitoring, digital health technologies, particularly wearable devices, offer significant potential for optimizing health management [25]. These technologies can serve as complementary clinical tools, supporting tasks such as early diagnosis and the objective monitoring of PD symptoms over time [26]. Wearable devices provide a discreet and user-friendly solution that not only reduces the burden on patients to remember and manually record information about their symptoms but also offers systematically organized data on symptom evolution [27]. This can be achieved by using digital biomarkers [28], which enable the objective assessment of disease progression and the impact of treatments or therapeutic interventions in chronic conditions such as PD.

### BioClite Project

BioClite (Digital Biomarkers for the Assessment of Motor Status in Parkinson’s Disease Patients With Clinical and Therapeutic Application) is a project funded by the Ministry of Science and Innovation of Spain (No. PID2021-123708OB-I00) that aims to enhance PD monitoring using wearable technology.

The BioClite project responds to the growing need for objective assessment tools to improve the management and long-term monitoring of PD using wearable devices, specifically smartwatches. By using this technology, the project aims to improve the accessibility of medical care and eliminate physical barriers between patients and health care providers.

Prior studies have used wearable devices to assess motor symptoms. In the study by Samà et al [29], bradykinesia severity was evaluated using a waist-worn device; in the study by Di Lazzaro et al [30], several wearable devices were used to assess several MDS-UPDRS tasks; Coates et al [31] focused on gait parameters using an inertial measurement unit placed on the lower back; and in the study by Antonini et al [32], multiple motor symptoms were assessed using a commercially available wearable device. According to a review on wearable devices for PD monitoring [28], the most commonly used wearables incorporate inertial measurement units, such as accelerometers and gyroscopes, typically embedded in smartwatches, wristbands, or worn on the lower back or ankles.

However, previous technology-based approaches have presented several limitations. Many required bulky or intrusive equipment, limiting their practicality for long-term or home-based use. Others were constrained to controlled environments, reducing ecological validity and overlooking symptom fluctuations in daily life. Furthermore, user compliance was often hindered by complex device management or lack of feedback. Importantly, some earlier systems did not sufficiently address data privacy concerns, raising potential risks regarding the handling of sensitive personal information, particularly when combined with the use of artificial intelligence models [33].

This study advances this field by implementing a dual-monitoring approach: guided exercises conducted in controlled environments, where participants perform specific tasks to capture motor patterns associated with PD, and free-living activities, where data are collected from participants in uncontrolled environments during their daily routines outside of clinical or laboratory settings without direct supervision and under natural conditions.

One of the daily-life activities is egg beating, an activity often identified by patients with PD as one that made them aware of their condition; this activity is performed under both supervised and unsupervised conditions. This approach not only allows for direct comparison between clinical and real-world data but also introduces an ecological, patient-centered perspective that is largely absent from previous research, which has typically relied on standardized clinical tasks. By including free-living monitoring, this methodology allows for continuous data collection without interrupting patients’ everyday activities, providing a more natural and unobtrusive experience compared to traditional methods.

Data labeling plays a crucial role in the project and is carried out using 2 main methods: algorithmic tagging, where custom-designed algorithms automatically analyze collected signals to identify significant motor events, and the use of external beacons and markers to link time points with contextual information, improving the accuracy of the tagging process. The combination of these approaches can support the collection of a comprehensive, annotated dataset that supports the development of algorithms for PD detection, status prediction, and personalized therapeutic strategies.

Through the collection of motor activity data, primarily in real-world environments, the project aims to develop digital biomarkers that provide valuable insights into PD symptoms, particularly tremors and bradykinesia. The protocol detailed in this paper describes a study designed to leverage digital biomarkers for PD detection, with the objective of distinguishing patients with PD from healthy individuals and enabling early-stage disease assessment. This approach allows continuous monitoring of symptoms and evaluation of therapeutic effects, thereby supporting personalized treatment strategies and improving the management of the disease.

The BioClite project contributes to more precise symptom tracking and the advancement of wearable technology in PD management by enabling continuous and unobtrusive monitoring, ultimately enhancing patient care and advancing scientific research in the field.

## Methods

### Inclusion and Exclusion Criteria

For the PD cohort, the main inclusion criterion is a diagnosis of PD based on the United Kingdom Brain Bank criteria. All participants in this group were recruited from Asociación Parkinson Madrid (APM), ensuring access to a well-defined population actively engaged in PD care and support.

To ensure a representative sample, efforts will be made to achieve a gender balance across the study population. In addition, participants must meet an age requirement ranging from 45 to 80 years, reflecting the study’s focus on middle-aged and older populations typically affected by PD [34].

The control participants are not selected based on strict age alignment with the PD patients, as both groups are recruited and analyzed concurrently. Instead, efforts will be made to ensure that the 2 groups exhibit comparable means and SDs with respect to age, such as pairing the recruitment of healthy controls with that of participants with PD or dividing participants into smaller age groups (eg, 55-60, 61-65 years) and ensuring a similar number of healthy controls and participants with PD within each group.

While this design choice may introduce variability, age will be included as a covariate in statistical analyses to account for its potential impact on the results. In addition, prioritizing inclusivity over strict matching allows a broader understanding of how the proposed biomarkers may generalize across diverse populations.

The common inclusion criteria established for participants with PD and control groups are as follows:

- Wear the study devices on the hand most affected by the condition and dominant (for patients with PD) or the dominant hand (for controls). In both cases, this should be the hand primarily used in the free-living activities.
- Be capable of performing prescribed exercises independently.
- Maintain daily functioning without dependence on caregivers.

Specific inclusion and exclusion criteria for the PD group have been designed to enhance better representative of the sample, given that the study focuses on the detection and initial stages of the disease, as participants with advanced PD often exhibit evident motor symptoms that are more readily identifiable; however, they may also experience impairments that could limit the feasibility or effectiveness of wearable monitoring technologies [35]:

- Eligible participants must have minimal or mild disease severity, defined by a Hoehn and Yahr scale score of ≤2.5.
- Participants must not experience motor symptom fluctuations throughout the day.
- Individuals who have undergone deep brain stimulation, used dopamine infusion pumps, or have conditions mimicking PD symptoms (eg, progressive supranuclear palsy, Lewy body dementia, multiple system atrophy, or other parkinsonian syndromes) are excluded.

### Recruitment Process

The recruitment of new participants begins with an initial contact, conducted either in person or by telephone, during which a concise overview of the study’s purpose and procedures is provided. If the prospective participant expresses interest and meets the inclusion criteria, they are invited to proceed with the next steps.

For participants in the PD group, an appointment is arranged at the APM, while control group participants attend a meeting at the Escuela Técnica Superior de Ingenieros Industriales at Universidad Politécnica de Madrid. During these sessions, the study’s objectives, procedures, and expectations are thoroughly explained to ensure participants have a comprehensive understanding of their role and responsibilities.

To formalize their participation, individuals are asked to review and sign an informed consent form in compliance with ethical guidelines and regulatory standards.

### Study Design

This study comprises 1 week of free-living monitoring of 20 participants with PD and 20 healthy control participants. The proposed number of participants in this study is comparable to those found in the literature for defining biomarkers in PD (mean 15, SD 6.8 patients with PD) [28,31,36-39] or for monitoring PD motor symptoms (mean 15.4, SD 4.5 patients with PD) [29,40-45]. Small samples are common in exploratory research with wearable sensors, particularly in populations considered vulnerable and hard to recruit such as individuals with PD. Such studies are important for early validation of methods and identifying key trends to inform future research. While the sample size limits detailed subgroup analyses, it is adequate for assessing general patterns and methodological feasibility. All participants will undergo the same experimental protocol, which includes the following:

- A baseline assessment to establish initial measures
- A 1-week period of unsupervised free-living activity monitoring to capture naturalistic data
- A final evaluation to reassess clinically and compare with baseline results

**Figure 1:**
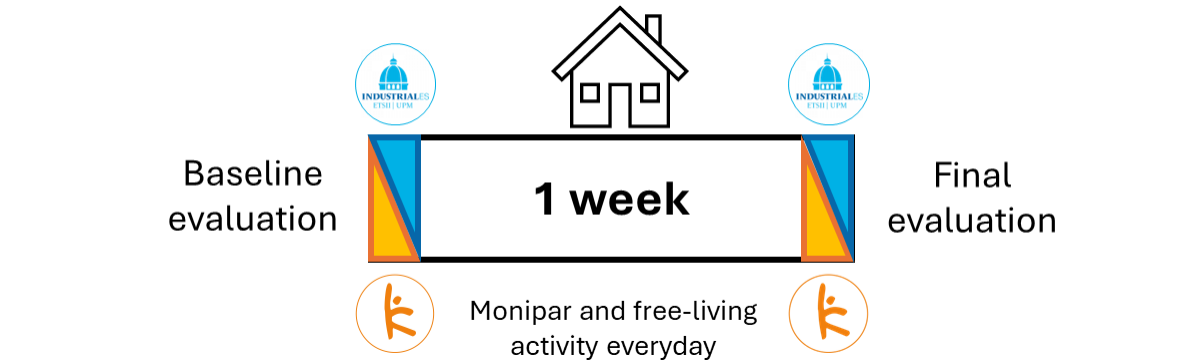
Overview of the experimental protocol of the study.

The study schema is illustrated in Figure 1, providing an overview of the structured protocol and its key phases.

Participants will engage in a series of standardized exercises and a structured free-living activity conducted at home. The findings from this study will establish a foundation for future research focused exclusively on assessing free-living activities without requiring patients to perform any predetermined tasks.

Upon recruitment, each participant begins the experimental protocol with a meeting at APM (for the PD group) or at the Escuela Técnica Superior de Ingenieros Industriales at Universidad Politécnica de Madrid (for the control group). During this visit, a baseline assessment of the participant’s motor symptoms is conducted using the Monipar set of exercises [46]. This set comprises 8 tasks specifically designed to evaluate various motor symptoms of PD.

Seven of these exercises are adapted from the MDS-UPDRS III scale: resting tremor amplitude (item 3.17), postural tremor of the hands (item 3.15), finger tapping (item 3.4), hand movements (item 3.5), pronation-supination movements of the hands (item 3.6), arising from a chair (item 3.9), and gait (item 3.10). The eighth exercise, proposed by the research team, complements the evaluation by addressing additional motor symptoms. These tasks are routinely used by neurologists in clinical settings, and participants are generally familiar with their performance, ensuring ease of implementation.

For the PD group, a specialized PD physiotherapist performs all the assessments using the MDS-UPDRS III scale to ensure consistency and reliability across participants.

In addition, to facilitate the transition from controlled clinical assessments to unsupervised free-living conditions, participants are required to perform a specific free-living activity. This activity involves beating eggs for 1 minute, a common daily task familiar to most individuals. The repetitive nature of this activity makes it particularly useful for evaluating bradykinesia [47,48] under free-living conditions. The selection of this activity was informed by consultations with nurse practitioners, patients, and therapists, all of whom stated its potential relevance and significance for assessing bradykinesia.

Following the baseline evaluation, participants proceed with the study in free-living, unsupervised conditions, maintaining their normal daily routines. They are required to wear the smartwatch throughout the day for 1 week, removing it only at night for charging. Vanmechelen et al [49] revealed that some participants feel discomfort using wearable devices, particularly at night, due to concerns about privacy and intrusion into daily life.

The smartwatch records accelerometer and gyroscope data during a designated 4-hour period each day. This recording window, determined by the participants at the study’s outset, accommodates their individual schedules and availability. Participants are expected to perform a series of tasks within this defined period, which will remain consistent throughout the 7 days of the study.

In the 4-hour recording period, each participant is asked to perform the Monipar exercises daily under free-living conditions and without supervision. Participants are encouraged to complete the full set of exercises each day, ideally at the same hour of the day.

Completing this set of exercises takes approximately 7 to 8 minutes daily, which does not significantly disrupt patients’ daily lives. Incorporating them as a routine task facilitates easier recall and adherence. In addition, participants will be required to perform the specific free-living activity of beating eggs for 1 minute each day. So, the participants will need to set aside 10 minutes each day to perform these 2 tasks.

In prior clinic-based studies [46], participants reported that they did not perceive the daily performance of these exercises as tedious or repetitive. However, modifications were implemented in the duration of each exercise to mitigate fatigue and to provide users with the flexibility to choose when to complete them. This adjustment is intended to enhance user compliance without compromising data quality.

In addition, participants will complete a daily paper questionnaire to help contextualize the 4-hour recorded sensor data. This questionnaire captures the timing of key activities such as breakfast, lunch, and dinner; rest periods (useful for assessing resting tremors); and medication intake. Participants are also encouraged to include any relevant comments for each day, such as forgetting to wear the smartwatch or if the device was completely discharged.

As participants are required to wear the smartwatch throughout the day, their physical activities, such as walking, running, or cycling, are automatically recorded and labeled by the device using its built-in apps [50]. Physical activities are characterized by step count, distance covered, and calories burned during each session. This automatic labeling allows any physical activity that occurs during the designated 4-hour signal recording period to correlate with the acquired accelerometer and gyroscope data, enhancing the contextual understanding of the recorded measurements.

After 1 week of free-living monitoring, participants return to APM for a final evaluation, conducted in the same manner as the baseline assessment. This includes performing the Monipar exercises under the guidance of the PD physiotherapist (for the PD group); completing the specific free-living activity; and filling out a questionnaire to assess the comfort and usability of the smartwatch, report any issues encountered, and provide additional observations or feedback.

### Devices Used in the Study

This study uses 2 different types of devices: a smartwatch (Samsung Galaxy Watch5) and a smartphone (Samsung Galaxy A14). The smartwatch serves as the primary measuring tool, responsible for recording all generated data throughout the study. In contrast, the smartphone is used to display instructions and label the proposed activities.

**Figure 2:**
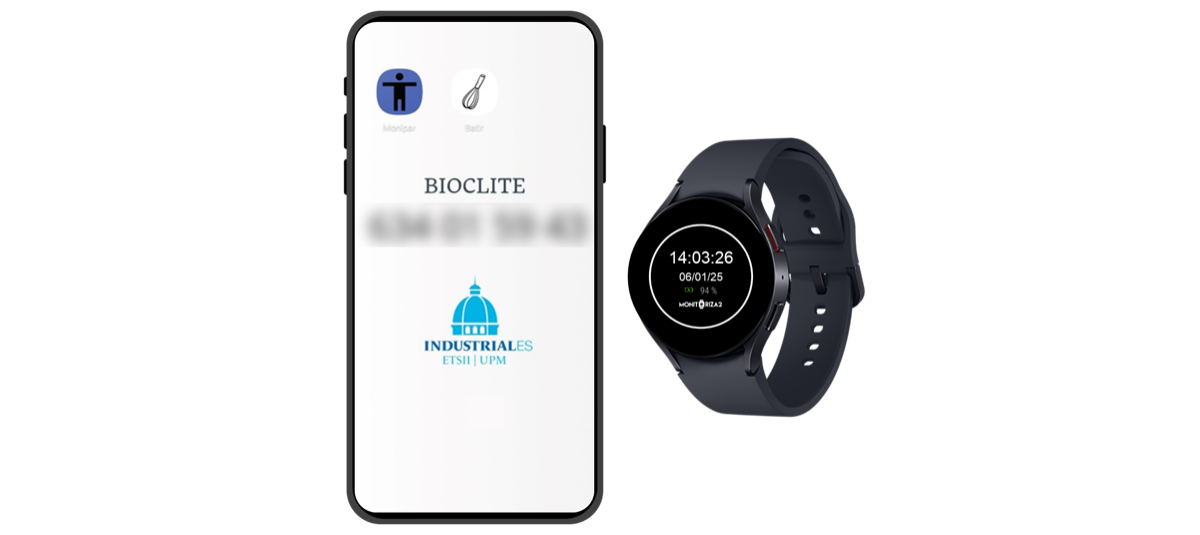
Devices used in this study: smartphone (left) and smartwatch (right).

Figure 2 illustrates the devices used in this study. The home screen of the smartphone displays 2 distinct icons representing the apps participants will use to complete the activities. A contact telephone number is displayed on the wallpaper (blurred for privacy purposes). In addition, the smartwatch screen is intentionally designed to display only essential information, such as the time, date, and battery status, to ensure user-friendliness and minimize distractions for participants.

The smartwatch incorporates an LSM6DS0 module, integrating a 3-axis digital gyroscope and a 3-axis digital accelerometer, which facilitate the recording of both acceleration and angular velocity. The smartwatch configuration was set to prevent the collection of unnecessary data, such as location, audio, or video, to ensure data relevance and privacy.

Sensor’s data are captured using an Android Wear OS custom app developed by the research team, configured to acquire data at a sampling rate of 50 Hz from each sensor. This frequency was chosen because it is well suited for analyzing PD motor symptoms, which typically occur within the 0 to 20 Hz range [51].

In addition, this custom app facilitates the scheduling of accelerometer and gyroscope signal recordings. In this study, a period of 4 hours per day is chosen for the free-living monitoring week. Continuous recording throughout the day is avoided for 2 primary reasons: to preserve participants’ privacy—because certain activities could be inferred from these sensor data—and to minimize battery consumption. With these considerations, data from the accelerometer and gyroscope are recorded for a duration of 4 hours, during which the participants must perform the proposed activities.

In addition, the smartwatch is responsible for collecting data related to the physical activity performed by each participant all day, obtained from the smartwatch’s dedicated apps.

In contrast, the smartphone functions as a user interface, facilitating participants’ execution of both the Monipar activity and the specific free-living activity. This was achieved through 2 dedicated apps designed to display and playback the instructions necessary for performing these tasks effectively.

While the apps were identical in functionality, each was designated for a specific task—one for the Monipar activity and the other for the specific free-living activity. This differentiation served 2 purposes: providing participants with the flexibility to perform each activity at their convenience and accommodating the additional preparation required for the specific free-living activity.

The smartphone synchronizes with the smartwatch; tags each activity performed; and, upon completion of the study with each participant, integrates the data recorded by the smartwatch with these labels. This process ensures accurate data labeling, aligning the sensor measurements with the corresponding activities for subsequent analysis.

The smartphone is set up to prevent the collection of unwanted data, such as location, audio, or video. In addition, participants are restricted from making calls or accessing apps unrelated to the study. To address potential participant inquiries, the phone number of a researcher is set as the device’s wallpaper.

**Figure 3:**
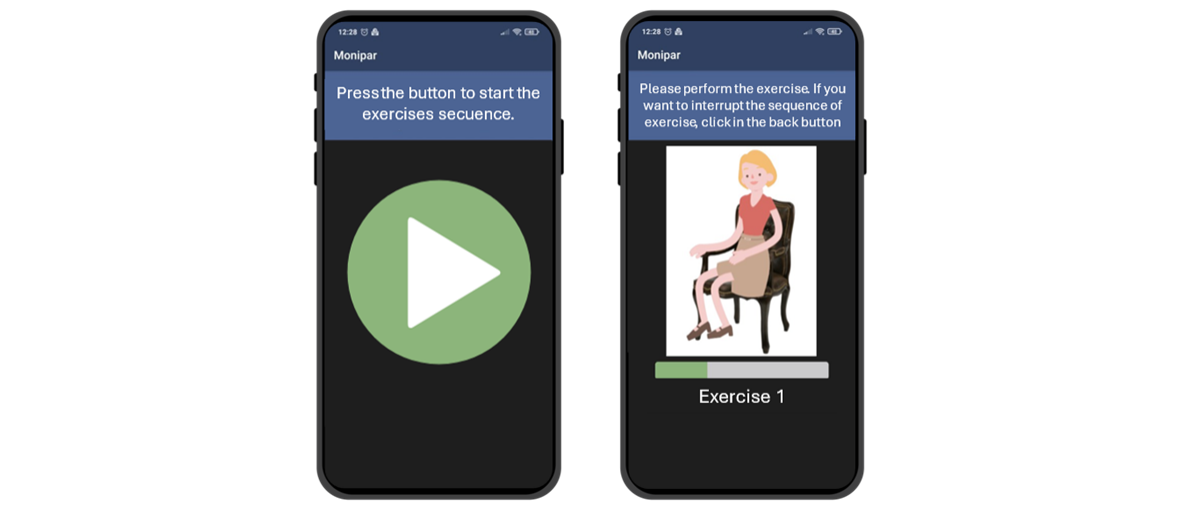
Interface of the app related to the Monipar activity: start screen (left) and activity screen (right).

**Figure 4:**
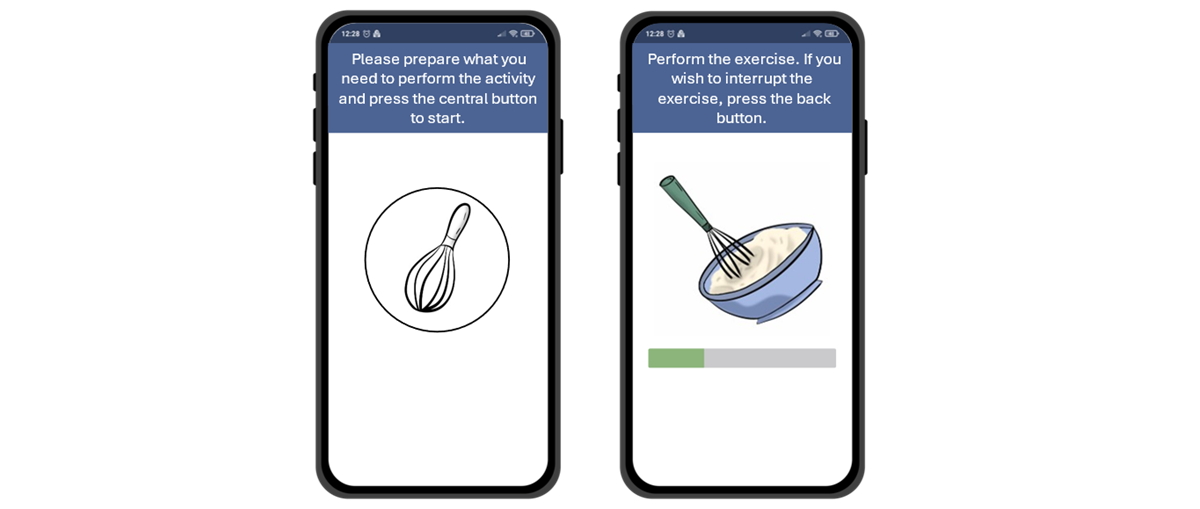
Interface of the app related to the specific free-living activity: start screen (left) and activity screen (right).

Figure 3 illustrates the interface of the Monipar app, while Figure 4 depicts the layout of the app for the specific free-living activity.

### Data Collection

The proposed system is designed for dual functionality, allowing use in both supervised clinical settings and unsupervised daily-life scenarios. Although all devices involved operate independently, they are temporally synchronized via Bluetooth, ensuring simultaneous data collection and seamless integration of information from multiple sources.

**Figure 5:**
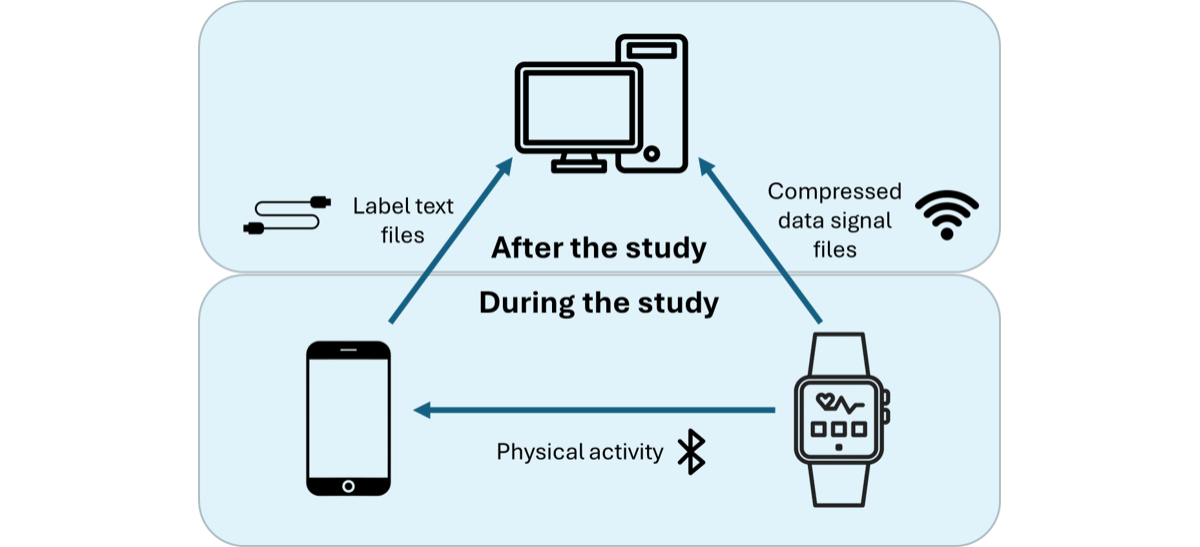
Structure of the data acquisition system.

The smartwatch serves as the primary device for recording data, while the smartphone facilitates the capture of time stamps for activity labeling. Data extraction and processing will occur after the study using a desktop computer. Figure 5 illustrates the architecture of the data acquisition system during and after the study.

The smartwatch worn by the participant acquires data from the accelerometer and gyroscope sensors for a duration of 4 hours each day. The data are recorded at a frequency of 50 Hz and stored in a text file in the device memory. Once the specified recording period is completed, the text file is saved in a compressed format (zip), and the original text file is deleted. This compression is done to prevent the risk of memory saturation on the device, as text files take up approximately 4 times the space of compressed files. This ensures that all daily data files are saved and stored on the device without any issues. These compressed files remain on the smartwatch until the device is retrieved at the final study visit.

Physical activity data will be collected using the device’s built-in apps, temporarily stored on the watch, and periodically transmitted via Bluetooth to the smartphone. The smartphone then retains this information until the study concludes.

The smartphone also generates text files containing time stamps that indicate the start and end of each exercise performed. As the exercises are tracked using 2 different apps, 2 separate label files are generated each day: one corresponding to the 8 exercises of the Monipar sequence and the other to the specific free-living activity. These text files are stored on the smartphone until the end of the study.

Upon completion of the study and the return of the devices by the participants, the collected data are extracted. The extraction process differs based on the type of wearable device, though in both cases, it requires the use of a computer. For the smartphone, text files containing the temporary labels are extracted via a direct cable connection to the computer. Conversely, for the smartwatch, the compressed data files from the inertial sensors are retrieved over a Wi-Fi connection, ensuring that both the watch and the computer are connected to the same network.

### Data Analysis

The resulting database from the data collection campaign will comprise signals from the 3 axes of the smartwatch’s accelerometer and gyroscope. These data will be divided into 3 distinct parts. First, it will include the evaluation of the sequence of 8 Monipar exercises, conducted both in supervised and labeled conditions following the MDS-UPDRS III scale, as well as in unsupervised free-living conditions. Second, it will encompass the performance of the specific free-living exercise, 1 minute of beating eggs, performed under supervised conditions at the association and under unsupervised free-living conditions. Finally, the database will contain the daily recording of 4 hours of free-living activity, accompanied by the physical activities performed during the day and participant-completed notebooks detailing activities such as eating, resting, and taking medication.

A comprehensive data analysis will be proposed to identify appropriate indicators and validate their effectiveness. Various scenarios will be proposed based on the collected data, providing insights into the potential applicability of these biomarkers in diverse clinical and therapeutic contexts.

A set of biomarkers will be defined for the identification of patients with PD by comparing their data with those of healthy participants. Another collection of biomarkers will be calculated to predict MDS-UPDRS scores in patients with PD using the labeled sessions, enabling a more detailed understanding of the relationship between the recorded signals and clinical evaluations.

In addition, a comparative analysis will be undertaken using the previous or new biomarkers between measurements collected in supervised clinical contexts and those recorded in unsupervised, free-living conditions. Similarly, the validity and performance of the proposed biomarkers will be examined across different age groups, given the motor variability present among various age ranges. This will help determine the consistency and reliability of the data across different contexts. Furthermore, the study will explore the correlation between the specific free-living exercise, which is strongly linked to bradykinesia, and the MDS-UPDRS exercises of the Monipar sequence. This correlation will be examined for both data collected at the APM and at the participants’ homes, providing insights into how specific activities reflect the motor symptoms in patients with PD across diverse settings.

**Figure 6:**
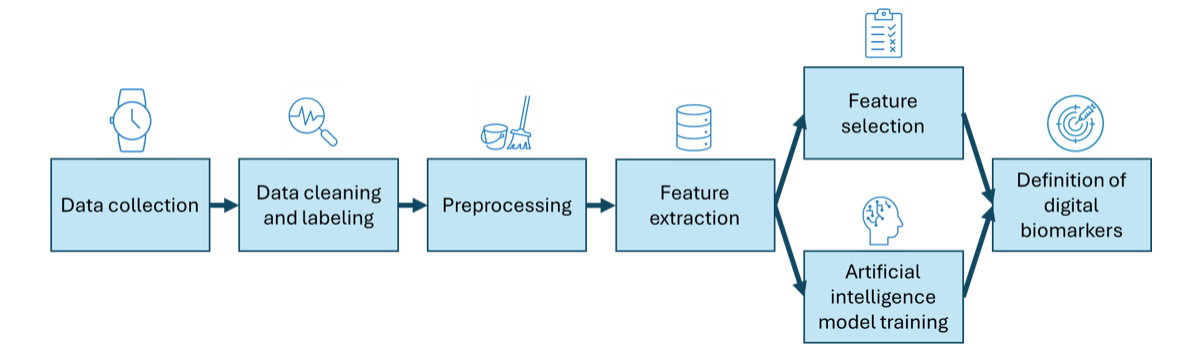
Data analysis pipeline.

To define these potential biomarkers, data analysis methods are developed and organized according to a data analysis workflow. As the entire database is based on the triaxial accelerometer and gyroscope signal, the pipeline will be applied to the whole database. Figure 6 shows the proposed pipeline. All data analysis will be conducted using Python (Python Software Foundation) and MATLAB (MathWorks).

First, the correct recording and collection of the expected data for each participant will be verified to ensure that all records are properly saved and stored. Once confirmed, the data will be labeled by combining the signals collected from the smartwatch with the activity tags generated by the smartphone. The 8 exercises of the Monipar sequence and the forced free-living activity will be tagged, with automatic tagging enabled by the synchronization between the 2 devices. However, a manual check will be conducted to ensure the accuracy of the start and end times for each tagged activity.

An individual analysis will be conducted for each participant’s data collected in unsupervised, free-living conditions to assess the reliability and trustworthiness of the signals. Although all participants will receive the same clear and standardized instructions, this step helps ensure that variations in guidance do not impact performance. To achieve this, the signals recorded in a supervised setting—reviewed by at least 4 experts (including a physiotherapist specialized in MDS-UPDRS and 3 other researchers or engineers to ensure correct exercise performance)—will be used as a reference for comparison with those recorded during free-living conditions. This will be done both for the set of exercises of the Monipar sequence and for the forced free-living activity.

Participants with corrupted, incomplete, or inaccurate data will be considered for inclusion only if they possess a complete supervised recording and at least half of the expected unsupervised data, ensuring sufficient data quality for reliable analysis.

The sessions conducted at the beginning and end of the study for the PD group will also be labeled, as these recordings are accompanied by evaluations performed using the MDS-UPDRS by a physiotherapist specialized in PD. It is crucial to highlight that the initial and final MDS-UPDRS assessments are performed by the same evaluator, who will evaluate each of the MDS-UPDRS exercises performed. This introduces 2 sources of subjectivity: one related to the evaluator’s individual judgment and the other inherent to the interpretation and use of the MDS-UPDRS scale, which is subjective by nature. To mitigate this potential bias, the evaluator had undergone standardized training procedures before assessments, and intrarater reliability will be monitored throughout the study to ensure consistency in scoring. These scores serve as the reference label for assessing the participant’s current condition and can be used to categorize individuals into groups based on the intensity of motor symptoms.

Once the data have been labeled, a preprocessing step will take place. During this phase, various actions will be performed depending on the specific analysis objectives. These actions include combining the 3 signals from each sensor using the Euclidean norm; applying filters (eg, low-pass, high-pass, or band-pass) to isolate specific frequency ranges; segmenting the signal into windows with or without overlap; resampling the signal to a different frequency; or transforming the time-domain signal into the frequency domain, for example. These preprocessing steps aim to prepare the data for further analysis, ensuring that the signals are in the optimal format for extracting meaningful insights.

Following the preprocessing phase, feature extraction will be conducted to identify and compile characteristics from the processed signals for further analysis. This process transforms raw data into a structured set of measurable attributes or features, facilitating analysis or modeling. The goal of this step is to reduce data complexity while preserving its most relevant and informative aspects. Extracted features may encompass statistical properties of the signals, such as mean, SD, or range, in both time and frequency domains [49]. In addition, they may be derived from mathematical computations or based on biomechanical analyses of the movements performed, targeting specific motor symptoms, including resting tremors, bradykinesia, or gait abnormalities.

Depending on the amount of extracted features, it may be beneficial to reduce their number, as a manageable and well-defined set of characteristics is essential for establishing reliable biomarkers.

Statistical and probabilistic analyses will be performed using the proposed features to identify potential biomarkers that correlate with the various PD symptoms being studied. These biomarkers may be consistent across all symptoms or distinct for each symptom, as well as for different activities.

Furthermore, artificial intelligence classification models will be developed using the proposed features. Given the medical context of this study case, it is crucial that the models are interpretable and explainable. To achieve this, machine learning algorithms such as random forests or decision trees will be used, as these models can be subjected to explainable artificial intelligence techniques. In addition, deep learning models will be incorporated to enhance the predictions made by the machine learning models. The most relevant features will be extracted from the models to identify digital biomarkers for motor symptoms of PD.

The evaluation of the classification models will be assessed through a confusion matrix, alongside metrics such as sensitivity, specificity, or accuracy. Other performance measures, such as the area under the receiver operating characteristic curve, could be considered to offer a more thorough evaluation of the model’s effectiveness.

In unsupervised measurements conducted at participants’ homes, the MDS-UPDRS score determined in the clinical setting can be used as a benchmark to evaluate its alignment with the results obtained from these unsupervised measurements. However, considering the intrinsic subjective nature of MDS-UPDRS labeling, unsupervised learning methods, such as association rules or clustering algorithms, will also be explored to analyze the assessment of the exercises performed to uncover data-driven patterns. This approach aims to provide a complementary perspective and reduce reliance on subjective evaluations.

In addition, the 4-hour period of free-living activity, recorded alongside the patient’s self-reported medication intake times, will be analyzed to assess the relationship between medication administration and the patient’s condition during the period of inertial signal recording. This analysis will involve segmenting the free-living activity data into periods before and after medication intake and comparing the evolution of the inertial signal and defined biomarkers during these periods.

All relevant features, both before and after model training, will be examined and used to define the biomarkers. These biomarkers will serve several purposes: differentiating patients with PD from healthy participants, assessing the MDS-UPDRS stage in both supervised and free-living conditions, and evaluating the ability of free-living activities to correlate with the disease stage of each patient. In addition, these biomarkers will help elucidate the relationship between the MDS-UPDRS assessment and free-living motor activities and assess the impact of surgical and therapeutic treatments and interventions on motor performance.

### Technical Support

At the beginning of their participation in the study, each participant receives a detailed set of instructions designed to facilitate understanding and adherence during the week of free-living monitoring. These instructions primarily consist of image-based guidelines to ensure clarity and ease of comprehension. These visual instructions are provided to each participant in printed form for convenient reference throughout the study. In addition, participants are given the phone number of a research team member, which is prominently displayed on their smartphone wallpaper and included in the instruction materials, allowing them to reach out with any questions or concerns during the study.

In addition, as participants in the PD group are members of the APM, they will receive personalized follow-up regarding their participation in the study. For individuals with whom in-person contact will not be possible, phone calls will be made—upon request—to remind them of specific aspects of the study. Furthermore, the final evaluation questionnaire will include a question regarding the participants’ willingness to receive further follow-up through alerts or calls. Once data collection is complete, a detailed analysis of each participant’s actual follow-up will be conducted to assess adherence to the study protocol.

### Ethical Considerations

The study protocol was approved by the ethics committee of the Universidad Politécnica de Madrid (reference numbers: BDPLEDEMDP-IPG-HUMANOS-20231030; BDPLEDEMDP- IPG-DATOS-20231030). The ethical guidelines established clear procedures and responsibilities for managing data protection before the processing of any personal data, ensuring full compliance with legal regulations and adherence to best practices in research. Personal data, including participants’ names and contact details, will be recorded on paper and securely managed by the designated data controller for the project. The data controller will pseudo-anonymize these records by substituting identifiable information with an alphanumeric code, according to the Spanish data protection agency.

This unique code will be used to log all other participant information, such as gender, age, educational level, profession, handedness, clinical data, and additional comments, into the project’s digital database. Researchers will have access only to this anonymized digital database and not to the original paper records containing identifiable information.

In addition, the database will include sensor data collected through a custom app developed by the research team, as well as health indicators obtained via smartwatch and smartphone apps. Tam et al [52] noted that many remote monitoring devices lack official medical certifications, raising questions about the validity and security of the collected data. However, the smartwatch used in this study has received several approvals from the United States Food and Drug Administration or the Conformité Européenne Marking for its use for functionalities such as sleep apnea detection and electrocardiogram monitoring.

To set up these devices, nonpersonal user accounts will be established, which will collect only physical attributes, such as height and weight, necessary for the accurate measurement of participants’ physical activity. Similarly, the devices will be configured to avoid the collection of GPS data, audio, video, images, or any other personal information that could potentially compromise individual privacy. These measures ensure the secure and ethical handling of sensitive participant data throughout the study.

Although previous studies have highlighted a lack of consensus among patients regarding data ownership and access, suggesting shortcomings in the communication and understanding of informed consent procedures [52], in this study, all participants will be thoroughly informed about the study details before their participation begins. Written informed consent will be obtained from each participant in accordance with Good Clinical Practice guidelines and International Conference on Harmonization standards. Furthermore, each participant will be provided with a copy of the signed consent form to allow them to review the terms at any time, thereby reinforcing transparency and autonomy in the data collection process.

Participation in this study is completely voluntary. Participants can withdraw at any time without providing a reason, and their decision will not affect the quality of care or services they receive from the organization. Upon withdrawal, any data previously collected from the individual will be excluded from the analysis, and no replacement participants will be recruited.

## Results

Recruitment for the study commenced in December 2024 and will continue until spring 2025. The findings from this study are anticipated to be published by late 2025 or early 2026. Furthermore, the results will be disseminated to national and international patient organizations, as well as shared with the public. The database generated will be published at European public repositories.

## Discussion

### Anticipated Findings

This paper presents the research protocol for the BioClite project at APM, a study focused on monitoring PD using smartphones and smartwatch sensors. The study aims to compile a dataset comprising 20 participants with PD and 20 healthy controls for assessing motor symptoms in individuals diagnosed with PD. The ultimate goal of the study is to facilitate the definition of digital biomarkers for assessing motor symptoms in patients with PD.

In recent years, the use of technologies such as smartphones [53], smartwatches [46], and other wearable sensors [32,54] for monitoring PD has garnered increasing attention. Furthermore, advancements in computational capabilities have enabled the development of algorithms capable of recognizing complex movements and patterns [25]. These developments highlight the considerable potential of portable solutions to effectively monitor PD and provide valuable insights into its progression and management.

The high variability in motor symptoms associated with PD necessitates the evolution toward continuous patient monitoring. In consequence, the BioClite study aims to bridge this gap by transitioning from controlled clinical assessments to follow-up in nonsupervised free-living environments, as high variability in motor symptoms associated with PD necessitates the evolution toward continuous patient monitoring.

This approach involves evaluating participants through the MDS-UPDRS exercise sequence and the specific free-living activity of beating eggs by analyzing accelerometer and gyroscope signals from a commercial smartwatch. These activities are performed in both supervised clinical settings and unsupervised free-living conditions. Although the inclusion of structured tasks introduces a semisupervised element, it achieves a necessary balance between methodological standardization and validity for initiating the transition to free-living contexts. This dual approach ensures a comprehensive assessment of motor symptoms under controlled conditions and in participants’ daily lives.

The proposed biomarkers will be designed for app in both supervised clinical context and unsupervised free-living environments, enabling neurologists and PD specialists to evaluate symptoms and monitor their progression across both contexts.

### Limitations

Despite its potential, the study has several limitations. The data collection period is limited to a week, which may not fully capture the long-term variability of symptoms in PD. In addition, the inclusion of unsupervised data introduces potential biases. Although efforts are made to standardize data collection (eg, tagging activities and monitoring data quality), variations in how participants perform tasks in a free-living environment cannot be fully controlled. Advanced signal processing techniques and robust filtering methods will be explored to mitigate these effects.

Another challenge relates to participant adherence. Not all participants may complete the entire protocol or provide the expected volume and quality of data. Variations in motivation, cognitive impairment, or practical barriers (eg, technical issues) could reduce the completeness of the dataset. Personalized follow-up and automated reminders will be used to improve adherence.

Future research will focus on exploring the use of this technology in long-term study periods to assess the potential use of the defined digital biomarkers for continuous and free-living monitoring between clinical visits. Moreover, the approach developed here can be adapted for use in clinical trials and therapeutic monitoring. Integration into digital health platforms may support large-scale longitudinal studies and personalized medicine approaches in PD.

Both the anonymized dataset and the results of the study will be made publicly available, supporting transparency, reproducibility, and further research in the field of PD digital health.

### Conclusions

This study represents an important first step toward the implementation of free-living monitoring for PD. It provides evidence that a simplified protocol, combining structured and daily-life tasks, can be used to detect PD and monitor motor symptoms in real-world conditions. The proposed methodology may support the detection of PD, as well as the evaluation of disease progression and therapeutic effects. The development of digital biomarkers from these data could substantially improve the clinical management of PD.

## Abbreviations

APM: Asociación Parkinson Madrid
BioClite: Digital Biomarkers for the Assessment of Motor Status in Parkinson’s Disease Patients With Clinical and Therapeutic Application
MDS-UPDRS: Movement Disorder Society-Unified Parkinson’s Disease Rating Scale
PD: Parkinson disease
